# A Randomized Crossover Trial of a Pressure Relief Technology (SensAwake™) in Continuous Positive Airway Pressure to Treat Obstructive Sleep Apnea

**DOI:** 10.1155/2017/3978073

**Published:** 2017-12-19

**Authors:** Richard K. Bogan, Charles Wells

**Affiliations:** ^1^SleepMed of South Carolina, Columbia, SC, USA; ^2^SleepMed of Central Georgia, Macon, GA, USA

## Abstract

**Objectives/Background:**

Improving adherence to CPAP devices is crucial to reduce the long-term morbidity associated with OSA. SensAwake is a unique pressure relief technology that aims to promptly reduce the pressure upon sensing irregular respiration indicative of wakefulness. The purpose of this study was to compare adherence and sleep-quality outcomes in patients treated by CPAP with and without SensAwake technology.

**Methods:**

Participants with moderate-to-severe OSA were randomized to use CPAP devices with or without SensAwake (4 weeks) before crossing over.

**Results:**

Sixty-five patients completed both arms of the trial. There were no statistically significant differences in CPAP adherence with or without SensAwake over the study period (SensAwake ON 272.67 ± 17.06 versus SensAwake OFF 289.09 ± 15.24; *p* = 0.180). SensAwake reported a significantly lower system leak, 90th percentile leak, and time spent with excessive (>60 L/min) leak. Subgroup analysis suggested a trend towards improved adherence in patients with moderate-to-severe insomnia when using SensAwake.

**Conclusions:**

Using SensAwake incurred benefit in terms of reduced leaks; however, SensAwake did not improve CPAP adherence or objective sleep quality. Further studies should investigate the accuracy of observed trends towards increased adherence using SensAwake among patients with OSA and insomnia.

## 1. Introduction

Obstructive Sleep Apnea (OSA) affects 2 to 4% of the middle-aged population and is characterized by periodic collapse of the upper airway during sleep. Consequences of the disease include excessive daytime sleepiness, reduced quality of life, and the development of longer-term complications such as hypertension, cardiovascular disease, and metabolic syndrome [1–5].

Continuous positive airway pressure (CPAP) is considered the gold-standard treatment for OSA [6]; however, despite its effectiveness, acceptance of and adherence to treatment is poor [7–9]. Causes for patients' noncompliance are likely to be for a number of reasons and probably includes factors related to disease and patient characteristics; treatment titration procedures; technological device factors and side effects; and psychological and social issues [10].

Improving adherence to CPAP devices is crucial to reduce the long-term morbidity associated with OSA. It is clear that side effects related to the CPAP device itself have a role in the lack of adherence in OSA patients with approximately two-thirds of CPAP users experiencing mask discomfort, nasal/pharyngeal dryness, or pressure intolerance. With increasing demands for the amelioration of CPAP side effects, there have been a number of comfort-related technological advances in CPAP equipment including nasal and face mask innovations, humidified systems, and pressure modality add-on options [10].

One possible source of reduced CPAP compliance among OSA patients may be the perception of excessive pressure during exhalation that the patient experiences during arousal from sleep. Reducing the pressure during wakefulness may improve comfort and potentially adherence without compromising therapy efficacy. SensAwake is a unique pressure relief technology developed by Fisher & Paykel Healthcare. The SensAwake modification aims to detect whether the patient is transitioning from sleep to wake by monitoring respiratory patterns [11]. Upon sensing irregular respiration indicative of wakefulness, the device promptly reduces the positive airway pressure to improve patient comfort to help facilitate a return to sleep [12].

The purpose of this study was to compare adherence and sleep-quality outcomes in patients treated by CPAP with and without SensAwake technology.

## 2. Methods

This study was a double-blinded (patient and physician), crossover randomized controlled trial (RCT) using block randomization to allocate patients to treatment order of CPAP using the Fisher & Paykel ICON Premo (a fixed-pressure device) with or without the SensAwake pressure relief technology.

Adult patients aged 18 to 75 years with moderate-to-severe OSA (AHI ≥ 10 per hour) were eligible for inclusion if they had a successful in-lab titration polysomnography (PSG); general sleep habits of at least seven hours' sleep on most nights and lights out at midnight or earlier; and fluency in both written and spoken English. Sleep-disordered breathing events (>10 s) were scored using the American Academy of Sleep Medicine (AASM) criteria: apneas defined as drop in airflow by ≥90% of baseline and hypopneas as drop in airflow by ≥30% of baseline with ≥4% oxygen desaturation. The patients were recruited from SleepMed of South Carolina (*n* = 61) and SleepMed of South Georgia (*n* = 9). Patients were excluded from the study if they had been prescribed and fitted with any PAP device in the past two years; if CPAP therapy was contraindicated; if they had any known factor or disease that might interfere with treatment adherence, study conduct, or interpretation of the results such as severe psychiatric disease, history of nonadherence to medical regimens, or unwillingness to comply with study requirements as determined by the principal investigator; or if they suffered from other significant sleep disorder(s) that would interfere with their ability to wear the CPAP device. Also excluded were patients who had been prescribed hypnotics and/or sedating medications; patients who had undergone any surgery of the mouth, nose, sinuses, or airways (for OSA, snoring, or otherwise) over the previous 12 months; patients who are required by the nature of their employment to not comply with therapy (e.g., truck drivers, airline pilots); or if the physician objected to their patient taking part in the study.

Random permuted blocks were used to randomize patients into the two treatment sequence groups. The randomization records were kept in a patient master log. The study coordinator set the device to the appropriate treatment arm according to the patient master log during the device setup visit. The physicians were involved during follow-up if there were any pressure related adverse events. Both the physician and the patient were blinded to the treatment. To ensure adequate blinding, SensAwake was turned ON in all devices and this setting displayed to the user. However, in the study arm labelled SensAwake OFF, the adjustable SensAwake pressure was set equal to the therapeutic CPAP. Thus, in the SensAwake OFF arm, SensAwake events were detected but did not modify the delivered pressure. In the study arm labelled SensAwake ON, the adjustable SensAwake response (i.e., pressure target when an awakening was detected) was set to 4 cmH_2_O. This was allowed to be increased to 6 cmH_2_O if the patient was uncomfortable.

Fifteen patients in each treatment arm were additionally randomized to receive sleep monitoring by a single night of PSG at the end of each treatment phase (at crossover and at the end of the study); the total number who received PSG was 23/30. Ten actigraph devices (ActiSleep Plus) were available for ambulatory sleep monitoring during the study period. A total of 18 patients used the actigraph device and had data recorded.

**Figure 1:**
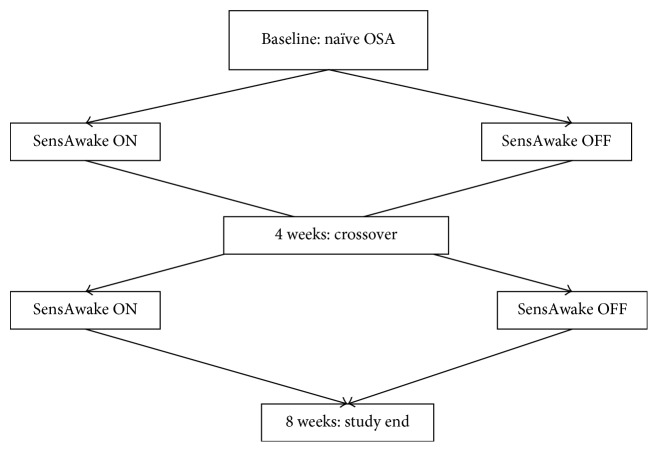


Data collection occurred at baseline, four and eight weeks (with follow-up phone calls at two and six weeks) with participants' crossing over at four weeks. Only data collected during the third and fourth weeks of use on each device was analyzed to ensure adequate washout between treatment arms. Participants were required to return to the study site to complete the crossover at four weeks and had to return again at the end of the eight-week study period (see Figure 1).

Existing data suggests that CPAP adherence without SensAwake is likely to average four to five hours (±1 to 2 hours) over a four-week period. For SensAwake to provide a clinically significant improvement in adherence, improvement by at least 0.5 hours was chosen as a predetermined endpoint. A pretrial power calculation indicated that if 70 participants were recruited and had a completion rate of 80% or more, the study would have sufficient power to detect a significant difference of 0.5 hours with greater than 80% power assuming a within subject standard deviation of 0.9 hours.

The primary outcome was adherence to treatment (hours per night obtained from F&P InfoSmart™ software). Secondary outcomes included objective sleep quality (including total sleep time, wake after sleep onset (WASO), and sleep efficiency measured using ActiSleep and PSG); subjective sleep quality (measured by the Epworth Sleepiness Scale (ESS), the Insomnia Severity Index (ISI), and sleep diaries); subjective treatment efficacy (measured using the Patient Global Impression of Change (PGI)); the impact of OSA on daily life (measured by the Fatigue Severity Scale (FFS) and the Short Functional Outcomes of Sleep Questionnaire (FOSQ-10)). Additional outcomes included complaints and participant-reported adverse events.

All analyses were performed using SPSS v22.0. Outcome data was compared between treatment arms using paired-sample* t*-tests, Wilcoxon signed-rank tests, and McNemar's Chi-square tests as appropriate to the data type. Post hoc analyses exploring the influence of insomnia on the relative treatment effects were conducted using a General Linear model. All statistical tests were considered significant when *p* ≤ 0.05. Results are reported as means ± standard error, unless otherwise indicated. Missing data points were handled by carrying the last observation forward; for example, if a participant had completed more than three weeks but less than four weeks on a treatment arm, the previous two weeks were used in the analysis.

Ethical approval was granted by Schulman Associates IRB (Study number FPH-SA13-01), and all patients gave written informed consent before commencement of the trial. The clinical trial was registered at https://www.clinicaltrials.gov (NCT01831258).

## 3. Results

### 3.1. Patients

Seventy patients were enrolled in the study and were randomized. Two withdrew from the study (both during the first arm with SensAwake OFF) and three were lost to follow-up. Data measured by actigraph and/or PSG was available for a subgroup of patients who were allocated to the additional device (ActiSleep) or who were randomized to undergo PSG. As such, the analyses presented are on a per-protocol basis. Sixty-five patients completed both arms of the trial. The mean age was 50.78 years (±11.49 years) and mean BMI was 35.93 kg/m^2^ (±7.97 kg/m^2^). The majority of patients (68%) were male and (69%) were Caucasian.

### 3.2. Adherence to Treatment

**Figure 2:**
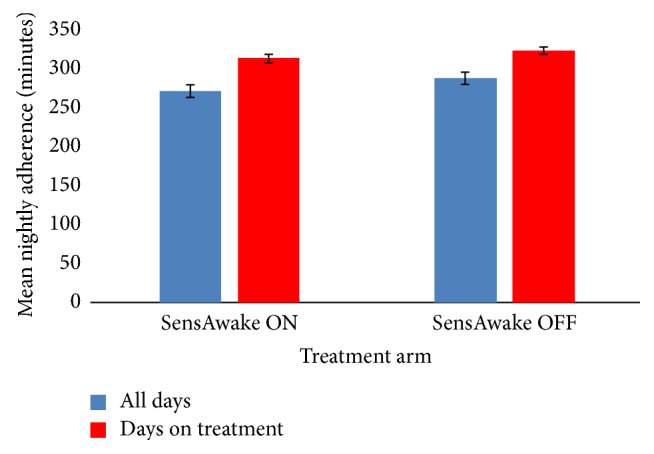
Adherence to CPAP devices with and without SensAwake.

There were no differences in the number of days that patients adhered to treatment; patients used the CPAP device for 12.1 days in the SensAwake ON group and for 12.2 days in the SensAwake OFF group (*p* = 0.72), 83.5% and 86.1% of the study period, respectively (Figure 2; Table 1).

Mean nightly adherence to treatment in minutes (Figure 2) was calculated from data downloaded from the CPAP device. There were no significant differences in adherence to CPAP for the average number of minutes on the days therapy was used (SensAwake ON 314.67 ± 14.6 versus SensAwake OFF 324.87 ± 12.83; *p* = 0.26) or for the average number of minutes used over the study period (SensAwake ON 272.67 ± 17.06 versus SensAwake OFF 289.09 ± 15.24; *p* = 0.18).

### 3.3. Objective Sleep Quality

In terms of leak, CPAP with SensAwake ON (41.72 ± 2.52) provided a significantly lower system leak (L/min) compared with SensAwake OFF (48.91 ± 3.01) with a mean difference of 7.19 L/min (95% CI: −10.51, −3.88; *p* < 0.001) (Table 1). SensAwake ON also provided a significantly lower 90th-percentile system leak (59.42 ± 3.328) compared with SensAwake OFF (64.341 ± 3.592) with a mean difference of 4.92 L/min (95% CI: −9.20, −0.65; *p* = 0.025). The time spent with excessive leak (>60 L/min) was also significantly reduced (SensAwake ON 18.85% ± 3.03 versus SensAwake OFF 26.38% ± 4.12; *p* = 0.006) with a mean difference of −7.53% (95% CI: −12.76, −2.30).

The Apnea Hypopnea Index (AHI) was slightly but significantly greater with SensAwake ON (5.18 ± 0.59) compared with SensAwake OFF (3.56 ± 0.46) using the data collected from the CPAP device (mean difference 1.62; 95% CI: 0.55, 2.69; *p* = 0.004). Patients in the SensAwake ON group fell just outside therapeutic targets (0 to 5 events/hour). The number of AHI events per hour was not found to be significantly different when determined from the PSG data (SensAwake ON: 4.58 ± 1.26 versus SensAwake OFF: 4.58 ± 0.90; *p* = 0.985). There were no differences in the length of apneas (SensAwake ON: 13.86 ± 0.54 versus SensAwake OFF: 13.19 ± 0.48; *p* = 0.158).

There were no statistically significant differences between CPAP devices with SensAwake ON and SensAwake OFF for any objective sleep-quality outcomes reported by actigraph or by PSG (Table 1).

### 3.4. Subjective Sleep Quality

Although CPAP with or without SensAwake improved outcomes on all subjective sleep-quality scales, there were no significant differences between SensAwake ON and SensAwake OFF for the ESS, FSS, ISI, PGI severity, or PGI change (Table 2). Both SensAwake ON and SensAwake OFF improved the FOSQ-10 compared to baseline (mean change of 4.63 ± 0.99 and 6.47 ± 0.90, resp.) but the FOSQ-10 was statistically higher in SensAwake OFF (*p* = 0.038).

### 3.5. Subgroup Analysis of Patients with Insomnia

Given that there were no differences in the primary outcome in all-comers, a post hoc hypothesis was tested that patients who are pressure intolerant may benefit from SensAwake, whereas those who report breathing easier with pressure may find it detrimental. Patients with OSA and insomnia are one group who has been shown to be less tolerant of CPAP pressure. Because the patients in this study completed the Insomnia Severity Index (ISI) as part of routine clinical practice, a subgroup analysis was possible to investigate the hypothesis that the 46% of participants with OSA and who were exhibiting signs of moderate clinical insomnia (ISI score ≥ 15 points) may benefit from SensAwake. This post hoc analysis showed a trend towards an increase in the average number of minutes on days of CPAP use with SensAwake in the initial four weeks on their first treatment with SensAwake ON (*n* = 18) compared to SensAwake OFF (*n* = 12), which did not reach statistical significance (SensAwake ON 322.89 ± 26.46 versus SensAwake OFF 282.25 ± 30.58). The patients in the SensAwake group reported a 40-minute greater use per night. Similarly there were no statistically significant differences in adherence for the average number of minutes used over the study period (SensAwake ON 304.78 ± 28.75 versus SensAwake OFF 238.42 ± 35.57); however, patients in the SensAwake group reported using the device for an average of 66 minutes longer.

After eight weeks of the crossover trial where the patients were exposed to both treatments, there were no significant differences in adherence to CPAP for the average number of minutes on the days therapy was used (SensAwake ON 281.85 ± 20.39 versus SensAwake OFF 291.74 ± 18.16; *p* = 0.657) or for the average number of minutes used over the study period (SensAwake ON 244.47 ± 23.48 versus SensAwake OFF 249.91 ± 21.39; *p* = 0.324). This suggested that, despite initial potential benefits in the insomnia group, changing therapy was not beneficial.

## 4. Discussion

CPAP with or without SensAwake reduced sleep-disordered breathing events to within generally acceptable targets for most outcomes and CPAP with SensAwake significantly reduced leak rates. Dungan et al. 2011 [13] investigated SensAwake in a study of similar design (crossover RCT) with patients receiving therapy for a single night in controlled conditions. Results showed that while OSA was adequately controlled with or without SensAwake, mean and 90% pressures were significantly lower. No other differences in sleep measures were reported between the two modalities. Because the study was conducted over a single night in a sleep laboratory, it was thought that findings may not relate to the actual ability of SensAwake to affect either acceptance or adherence to AutoCPAP therapy over the medium to long term; the authors suggested that further home-based studies were needed to evaluate any longer-term influence of SensAwake, particularly on wake after sleep onset (WASO). This study addresses some of these suggestions, it was home-based and each modality was used for four weeks, but no differences between groups were found for WASO or other objective sleep-quality measures as expected.

Air leaks are problematic for OSA patients for several reasons. Significant mask leak can reduce the ability of CPAP to maintain the prescribed pressure and prevent titration algorithms from properly responding to the required pressure changes. Significant leak can also disturb a patient's sleep quality by blowing excess air into the eyes or making noises that can cause additional arousals from sleep. For patients with mouth leaks, the increased airflow through the upper airway can cause nasal and pharyngeal dryness. In addition, significant system leaks have been found to have important associations with the development of treatment-emergent central apnea [14]. Considering the significant reductions in system leak, excessive leak (>60 L/min), and time with excessive leak when the SensAwake device was turned on, it is conceivable that SensAwake offers real benefits for OSA patients compared to the use of CPAP machines without the SensAwake device. This study did not show a reduction in the number of awakenings; however, the number of patients (*n* = 18) who had data recorded using an actigraph was small and may be subject to bias. Further studies recruiting larger numbers of participants could investigate whether efforts to eliminate leaks using SensAwake will lead to a reduction of awakening and the resolution of central apneas.

Despite the fact that SensAwake ON did not prove significantly more beneficial then SensAwake OFF in terms of objective and subjective sleep-quality outcomes, CPAP treatment overall improved subjective sleep quality.

Insomnia is prevalent among OSA patients; a recent review including 10 studies estimated a prevalence of 39 to 58% [15]. The existence of insomnia has been shown to negatively affect CPAP compliance [16, 17]. It is proposed that insomnia patients are preoccupied with external factors that may be perceived as a threat to sleep, which results in a higher wake after sleep onset, and which may be further exacerbated by the presence of CPAP [16]. Post hoc subgroup analysis showed that using SensAwake in addition to CPAP could be beneficial in OSA patients with signs of clinical insomnia; patients with signs of insomnia in the SensAwake ON group used CPAP for an average of 40 minutes longer on the days CPAP was used and for an average of 66 minutes longer during the first four-week treatment period. When comparing across the whole eight-week study period these differences diminished, suggesting that order does affect treatment compliance. The trend towards increased adherence among this population warrants further investigation.

Limitations of this study include the lack of intention-to-treat analysis, the small sample size, and the limited access to actigraph and PSG measures. Per-protocol analyses could not be avoided due to patient withdrawals/dropouts. The authors were unable to conduct PSGs on the full sample of patients for a variety of reasons and although randomizing available patients to PSG helped to limit bias, the number of patients for whom data was collected was small and may not be precise. Similarly, the number of actigraph devices available meant that data was not recorded for all participants.

There were no differences in the rates of adherence between CPAP with and without SensAwake. Using SensAwake incurred benefit in terms of reduced leaks; however, SensAwake did not improve objective sleep quality. Further studies should investigate the accuracy of observed trends towards increased adherence using SensAwake among insomnia patients.

## Figures and Tables

**Table 1:** Objective sleep quality in CPAP devices with and without SensAwake.

	SensAwake ON (mean ± SE)	SensAwake OFF (mean ± SE)	Mean difference (95% CI)	*p* value
*CPAP data *(*n* = 61)				
Number of days device used	12.1 ± 0.4	12.2 ± 0.3	−0.15 (−0.97, 0.68)	0.723
Average minutes used, days on therapy	314.7 ± 14.6	324.9 ± 12.8	−10.2 (−28.9, 7.9)	0.264
Average minutes used, all days	272.7 ± 17.1	289.1 ± 15.2	−16.4 (−40.6, 7.8)	0.180
AHI (events/hour)	5.2 ± 0.6	3.6 ± 0.5	1.6 (0.6, 2.7)	0.004
Length of apnea (Sec)	13.9 ± 0.5	13.2 ± 0.5	0.7 (−0.3, 1.6)	0.158
System leak (L/min)	41.7 ± 2.5	48.9 ± 3.0	−7.2 (−10.5, −3.9)	<0.001
90th-percentile system leak (L/min)	59.4 ± 3.3	64.3 ± 3.6	−4.9 (−9.2, −0.7)	0.025
Time with excessive leak (%)	18.8 ± 3.0	26.4 ± 4.1	−7.5 (−12.8, −2.3)	0.006

*Actigraph data *(*n* = 18)				
Total sleep time (min)	382.7 ± 13.5	378.9 ± 14.7	3.8 (−18.5, 26.1)	0.721
Sleep latency (min)	3.1 ± 0.7	2.4 ± 0.7	0.6 (−0.8, 2.1)	0.370
Sleep efficiency (%)	89.3 ± 1.2	89.0 ± 1.5	0.3 (−1.7, 2.3)	0.777
Wake after sleep onset (min)	42.6 ± 5.5	43.4 ± 6.2	−0.8 (−8.3, 6.8)	0.835
Number of awakenings (no.)	11.2 ± 1.0	11.6 ± 0.9	−0.3 (−1.8, 1.2)	0.655

*PSG data *(*n* = 23)				
Total sleep time (min)	459.4 ± 3.9	451.2 ± 6.6	8.2 (−3.9, 20.3)	0.175
Wake after sleep onset (min)	79.6 ± 11.1	83.5 ± 9.7	−3.9 (−21.3, 13.4)	0.642
Duration of REM (min)	83.5 ± 7.2	84.4 ± 7.4	−0.9 (−14.8, 13.0)	0.895
Duration of NREM (min)	299.6 ± 9.8	294.0 ± 9.4	5.7 (−10.3, 21.6)	0.469
Sleep efficiency (%)	83.2 ± 2.4	83.5 ± 2.0	−0.3 (−4.6, 4.1)	0.889
AHI (events/hour)	4.6 ± 1.3	4.6 ± 0.9	−0.02 (−2.2, 2.2)	0.985

CPAP: continuous positive airway pressure; AHI: Apnea Hypopnea Index; CI: confidence interval; PSG: polysomnography; REM: rapid eye movement; NREM: nonrapid eye movement.

**Table 2:** Subjective sleep quality in CPAP devices with and without SensAwake.

	*n*	Baseline	SensAwake ON mean change from baseline (mean ± SE)	SensAwake OFF mean change from baseline (mean ± SE)	Mean difference in change from baseline (95% CI)	*p* value
ESS	65	10.7 ± 0.6	−3.8 ± 0.6	−4.3 ± 0.6	0.5 (−0.3, 1.2)	0.227
FSS	64	36.2 ± 1.8	−6.9 ± 1.6	−8.0 ± 1.9	1.1 (−1.5, 3.7)	0.409
ISI	63	14.0 ± 0.7	−6.2 ± 0.8	−6.7 ± 0.7	0.5 (−0.7, 1.7)	0.421
PGI severity	64	1.0	4.3 ± 0.2	4.4 ± 0.2	−0.2 (−0.5, 0.2)	0.400
PGI change	64	1.0	4.2 ± 0.2	4.3 ± 0.2	−0.1 (−0.4, 0.2)	0.484
FOSQ	62	28.1 ± 0.8	4.6 ± 1.0	6.5 ± 0.9	−1.8 (−3.6, −0.1)	0.038

ESS: Epworth Sleepiness Scale; FSS: Fatigue Severity Scale; ISI: Insomnia Severity Index; PGI: Patient Global Impression of Change; FOSQ: Short Functional Outcomes of Sleep Questionnaire.
